# The War and German Child-Life

**Published:** 1919-05-03

**Authors:** 


### THE WAR AND GERMAN CHILD-LIFE.

THE mere figures of recently-published reports upon infant welfare in enemy countries during the past years of war do not tell of a more severe setback to the birth-rate or a greater rise in mortality among children than would be expected in a land the people of which passed through such oscillating phases of careless jubilation, frenzied effort and ultimate collapse as were seen in the case of the German Empire. It is true that the first three years alone resulted in the relative loss of at least two million children who would have been born in normal times, and that, taking particular years, 40 per cent. fewer babies were born in 1916 than in 1913, but, reading between the lines, the fact which appears so surprising, when connected with a Government which had prepared for years for the most terrible war of all time, counted its costs and laid plans intended to meet either the most trivial or the greatest emergencies, is that at the outbreak of hostilities apparently the minimum provision had been made for the strain on the country's infant life.

At the outbreak of hostilities such welfare work as was normally conducted suffered almost extinction through the pressing needs of the military and from Governmental indifference, and had it not been that a number of voluntary societies sprang rapidly into being the needs of the child-life of Germany would have been left utterly unheeded—throughout all the momentous days of 1914. As it was, the turn of events early in the following year brought an abrupt realisation that it was to be a long war, a war of exhaustion and that every life was of value. At once the Imperial Government turned to grapple with the problem, and fortunately for them private enterprise had already successfully started. Gradually governmental methods and organisation took over all previously-formed societies and decided the distribution and character of maternity or other grants, welding the whole into a direct extension of the municipally-controlled insurance societies and the sickness insurance laws. All was done with the harshest methods of discipline, routine and utilitarianism. Even in Germany this mode of action raised a storm of criticism from those who held that such matters were best dealt with directly through the trained staffs of the welfare centres—namely, the only people qualified to judge particular situations and needs. It was impossible for a generalised semi-military, semi-municipal organisation to create a series of grants and relief methods generally applicable to the whole country. Nevertheless, as in all things at that time, imperialism prevailed, and a set of regulations was formulated in 1915 by an Imperial Association for the Care of Infants. Previously acting welfare centres were aided and provided with funds for extension, but, whatever the proficiency of their staffs, the entire power lay in municipal hands, and the administration was conducted upon routine lines. Altogether about 800 centres arose among the areas of 550 local authorities. To take instances of the general methods employed, special importance was attached and special grants made to women who breast-fed their own children. The care of illegitimate children became increasingly urgent; taking the whole of the German Empire the proportion of such births was about 10 per cent., but in certain districts the rate became very much higher. All forms of guardianship of these children were exercised with the greatest strictness, and thus for the individual case the situation probably improved. Grants were made to unmarried mothers provided that simple proof was forthcoming that the father served with the army; in fact, a letter to the mother written from the battle front was taken as sufficient evidence. At first attention was directed only to the care and compulsory presentation for inspection of all children from birth to five years old, but in practice it appears that both inspection and distribution of relief were made by authorities of no specialised training.

Later there developed antenatal clinics, and as the urgency of the economic situation increased so benefits for and supervision of this branch compelled more and more attention, until in the last year of war there is no doubt that an enormous effort was being made to encourage and foster the child-life of the nation. On the whole, in this very necessary welfare campaign, Germany has favoured the modern lines of caring for the child in its own home, but when the women became, in increasing numbers, compelled to leave for the fields and factories, then more consideration was given to the institutional method. A series of establishments of somewhat crude efficiency was set up in which a great number of babies could be left while the mothers were at work, but relatively few were taken permanently away from the maternal care.

